# Integrative, In Silico and Comparative Analysis of Breast Cancer Secretome Highlights Invasive-Ductal-Carcinoma-Grade Progression Biomarkers

**DOI:** 10.3390/cancers14163854

**Published:** 2022-08-09

**Authors:** Stavroula L. Kastora, Georgios Kounidas, Valerie Speirs, Yazan A. Masannat

**Affiliations:** 1 Institute of Medical Sciences, School of Medicine, Medical Sciences and Nutrition, University of Aberdeen, Aberdeen AB24 2ZD, UK; 2 Breast Unit, Aberdeen Royal Infirmary, Aberdeen AB25 2ZN, UK

**Keywords:** bioinformatics, comparative analysis, IDC, biomarkers

## Abstract

**Simple Summary:**

Globally, breast cancer (BC) is the most frequently diagnosed cancer in women. Rapid advances in omics technologies have enabled the identification of biomarkers across various malignancies, including BC. The aim of this study was to enable identification of novel secreted biomarkers that may indicate progression to high-grade BC malignancies and therefore predict metastatic potential. We conducted a comprehensive systematic review to identify eligible secretomic datasets. This study identified putative predictors of IDC grade progression and their association with patient mortality outcomes, namely, HSPG2, ACTG1, and LAMA5. The present study highlights novel putative secretome biomarkers that may provide insight of the tumor biology and could inform clinical decision making in the context of IDC management in a non-invasive manner.

**Abstract:**

Globally, BC is the most frequently diagnosed cancer in women. The aim of this study was to identify novel secreted biomarkers that may indicate progression to high-grade BC malignancies and therefore predict metastatic potential. A total of 33 studies of breast cancer and 78 of other malignancies were screened via a systematic review for eligibility, yielding 26 datasets, 8 breast cancer secretome datasets, and 18 of other cancers that were included in the comparative secretome analysis. Sequential bioinformatic analysis using online resources enabled the identification of enriched GO_terms, overlapping clusters, and pathway reconstruction. This study identified putative predictors of IDC grade progression and their association with breast cancer patient mortality outcomes, namely, HSPG2, ACTG1, and LAMA5 as biomarkers of in silico pathway prediction, offering a putative approach by which the abovementioned proteins may mediate their effects, enabling disease progression. This study also identified ITGB1, FBN1, and THBS1 as putative pan-cancer detection biomarkers. The present study highlights novel, putative secretome biomarkers that may provide insight into the tumor biology and could inform clinical decision making in the context of IDC management in a non-invasive manner.

## 1. Introduction

Globally, breast cancer (BC) is the most frequently diagnosed cancer and the leading cause of cancer death in women. The estimated number of incident BC cases worldwide exceeded 2.2 million in 2021 [1]. Upon BC diagnosis, it is critical to appropriately define the disease to guide treatment options and clinical management. A variety of BC histological types have been identified, each with distinct microscopic appearance and molecular behavior. Invasive ductal carcinoma (IDC) is the most prevalent type of invasive BC, which accounts for 70 to 80 percent of all invasive carcinomas, followed by invasive lobular carcinoma (ILC) and mixed ductal/lobular carcinoma (MDLC). Other histological BC entities include metaplastic, mucinous, tubular, medullary, and papillary carcinomas. Ductal carcinoma in situ (DCIS), on the other hand, refers to a wide spectrum of precancerous lesions, which are confined to the breast ducts. DCIS, depending on initial tumor size, nuclear grade, presence, and extent of comedo necrosis, may progress to invasive disease. Nonetheless, the rate at which this progression may occur varies greatly across literature reports, ranging from 13.7% to 70% [2]. Invasive carcinoma can be found in patients with DCIS of all grades [2]. Collectively, literature findings suggest that, while high-grade DCIS may advance more quickly to invasive disease, all grades have a significant potential to progress. Furthermore, some DCIS may never progress to invasive disease, presenting a treatment dilemma for clinicians. Therefore, identifying non-invasive, specific, and sensitive biomarkers that may be utilized in conjunction with clinical and radiological findings to predict progression risk remains critical.

Through molecular profiling, further BC subtypes have been identified, including luminal subtypes (A and B), which represent the most common subtypes of BC and account for most estrogen (ER)-positive BCs. These tumors bear a significant histological resemblance to the luminal epithelium of the breast and typically express cytokeratins 8 and 18. Another prominent BC subtype includes the human epidermal growth factor receptor 2 (HER2)-enriched lesions. These lesions, which account for 10 to 15% of all BCs, and are characterized by high expression of HER2. These tumors are often ER and progesterone receptor (PR) negative. Lastly, the final category includes basal BC subtypes, the majority of which represent triple-negative breast (negative for ER, PR and HER2) malignancies [3,4,5].

Nearly six decades ago, Jensen and Jacobson [6] discovered the importance of steroid hormone receptors in driving breast carcinogenesis and their contribution to the progression and metastatic niche, demonstrating that radiolabeled estrogens preferentially concentrate in the estrogen-influenced target organs of both animal and human breast cancers, suggesting the presence of a core receptor, the ER. Since then, it has also become apparent that most BCs were dependent upon estrogen and/or progesterone for growth and progression. As a result, BC overexpression of ER and PR receptors has been used to develop hormonal antagonists in the form of endocrine therapy and to predict which patients would benefit from this. Of note, PR status is heavily dependent on ER; therefore, PR does not appear to have independently predictive value, especially when the ER status is known [7]. For ER- or HER2-positive malignancies, a variety or neoadjuvant and adjuvant chemotherapeutic and endocrine strategies exist to enable surgical resection of larger tumors extend while reducing the likelihood of progressive metastasis. On the contrary, there are no approved targeted treatments for TNBC, although immunotherapy (in combination with chemotherapy) is available for patients with advanced TNBC that expresses programmed cell death ligand 1 (PD-L1) [8].

Additional biomarkers are becoming increasingly utilized clinical practice, notably Ki-67 [9]. This has been extensively studied especially in the context of early BC and has been found to be an independent prognosticator of relapse and survival in both node-positive and node-negative disease [10,11,12]. With the drastically expanding knowledge of BC intracellular and extracellular profiles offered by large-scale omics datasets, more putative, predictive biomarkers, such as PIK3CA and p53 genomic mutations, overexpression of E-cadherin and catenins, tissue inhibitors of metalloproteinases, prostate-specific antigen, tissue factor, and urokinase plasminogen activator (uPA) protein abundance, have been highlighted either in the context of patient survival or response to chemotherapy [13,14,15,16,17,18,19,20,21,22,23].

Proteins expressed by a cell and subsequently secreted into the extracellular space constitute the cellular secretome [24]. Secretome analysis has emerged because of improvements in robustness and specificity of proteome isolation and analysis, which has been primarily used in tumors with endocrine components, such as lung and pancreatic malignancies, with great success in identifying novel biomarkers [25,26,27]. Similarly, the number of BC secretome datasets has been steadily increasing in the literature [28,29]. Whilst providing a wealth of information, individual datasets always harbor the risk of bias, either due to inherent experimental biases inevitably introduced by cell lines employed, equipment, or laboratory -and human- specific factors [30]. Therefore, pooling of BC secretome datasets provides an integrated, bioinformatic approach for decreasing individual study bias and highlighting BC biomarkers that extend beyond cell line specific hormonal receptor status, thereby having a broader clinical applicability.

The present work is the first integrative analysis of BC in comparison to other adenocarcinoma secretomic data. The aim of the study was to enable identification of novel secreted markers that could signal progression from in situ disease to invasive malignancy or progression of invasive disease to high-grade malignancies and therefore predicting metastatic potential. A total of 26 complete datasets were normalized, yielding 29 novel markers, which are hierarchically secreted in a stepwise manner between grade I and II to grade III BC malignancies. Correlation of biomarker levels with patient overall survival was also explored. This list of bioinformatically validated secreted proteins in BC may serve as potential biomarkers or as targets for novel therapeutic approaches to the disease.

## 2. Materials and Methods

### 2.1. Systematic Review

A systematic literature review was conducted to identify studies with original BC secretome datasets and, comparatively, studies presenting novel secretome datasets for other cancer types. Two independent reviewers (S.L.K. and G.K.) searched the literature for relevant studies up to 15 May 2022 on three databases: EMBASE (Ovid), MEDLINE (Ovid), and Web of Science. The references of the included studies were scrutinized for additional relevant studies. Search limitations included samples of human participants or human cell lines, and English language articles with full-text available.

The following search term was used in OVID for breast cancer: (Breast cancer OR Breast malignanc*AND Secretome OR secretome proteomic* OR secretome profil* OR secretome mass spectrometry OR secretome spectrometry).mp. [mp=ti, ab, hw, tn, ot, dm, mf, dv, kf, fx, dq, nm, ox, px, rx, an, ui, sy]. Equally, to identify secretome studies for other cancers, we used the following search term: (Cancer OR malignanc* AND Secretome OR secretome proteomic* OR secretome profil* OR secretome mass spectrometry OR secretome spectrometry).mp. with the same restrictions. After removing duplicates, citations were screened by title, abstract, and full text appraised to determine their eligibility by S.L.K. and G.K. Only studies utilizing human cancer cell lines, primary cell culture, or patient-derived samples were included. Equally, only studies with complete datasets (registered on ProtBase with accession number or uploaded as associated Supplementary Materials) were included in the analysis. Data were normalized against the control strain protein levels as described in each individual study (log2 fold, e.g., log2 fold change in comparison to wild-type control where fold signifies the ratio mutant (cancerous)/wild (control) type and original studies *p*-values) and combined in MS Excel. The proteins identified by each study included, were mapped to the same protein symbol, and those identified simultaneously in four or more studies (or ≥50% of cancer datasets) were selected for downstream analysis. In addition to protein levels per experiment, the following data were extracted per study: Author, Date, Title, ProtCode, Cell line, and Equipment. Cell lines were annotated according to pathology and clinical characteristics [type, grade, hormone receptor status (positive/negative)].

### 2.2. Data Processing, Visualization, and Statistical Analysis

Network construction was performed with Cytoscape V.3.7.2 freeware [31]. Venn diagrams were constructed using Venny (v. 2.1.0) online freeware [32] and the online platform for Venn diagram generation of Bioinformatics and Evolutionary Genomics [33]. The biological function of the genetic targets was analyzed with ClueGo V 2.3.3 plugin for Cytoscape [34]. Statistical analyses were performed using the Bonferroni-Holm Step Down approach, and biological function clusters were selected and visualized in a pie chart only if they met the *p*-value 0.001 criterion. Further settings included the following: (1) GO term fusion option was selected, (2) statistical options were enrichment/depletion (two-sided hypergeometric test) with Bonferroni-Holm step down approach, (3) leading group term was based upon calculated kappa score, and (4) only pathways with *p* < 0.001 were considered.

Heatmaps were generated with Morpheus online freeware [35], and statistical analysis was performed using GraphPad Prism (v. 9.3), a commercially available statistical program that was used for the statistical analysis. A *p*-value < 0.05 was considered statistically significant. The Human Protein Atlas was accessed, and survival data of breast cancer patients overexpressing the encoding genes of the proteins constituting central secretome cluster were downloaded [36]. Kaplan-Meier survival plots and adjusted risk ratio (HR) with the corresponding 95% confidence interval and log-rank *p*-value (*p*) were determined by Cox univariate regression analysis using GraphPad Prism (v. 9.3).

Protein/protein physical subnetwork identification (Homo sapiens) was conducted with the STRING v.11 [37]. Minimum interaction confidence was set at medium [0.4] and limited to interactions only between the input dataset and a maximum of 10 interactors. Only evidence stemming from experiments or databases were considered. Publications generating STRING interactions were then manually curated to delineate the nature of interaction. Pathway reconstruction was conducted with BioRender online platform (academic license).

## 3. Results

### 3.1. Study Characteristics

**Figure 1:**
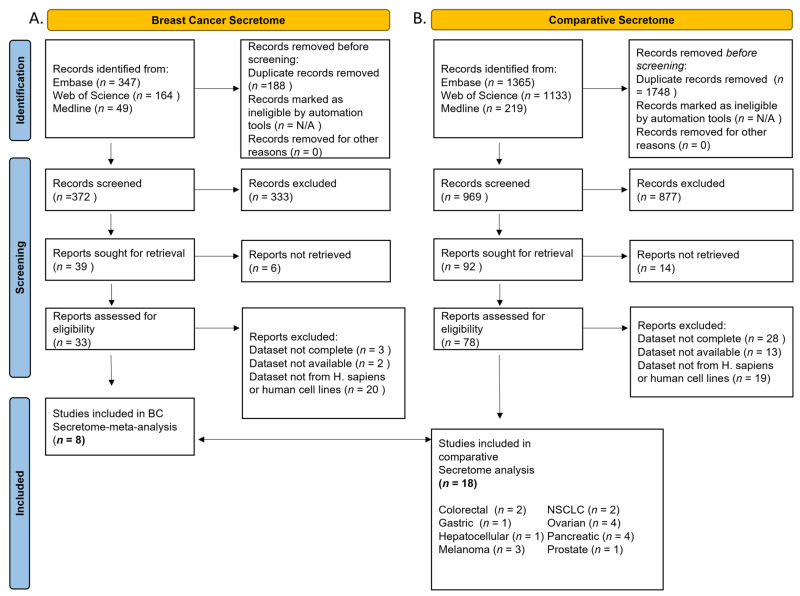
Identification and screening flowchart of BC vs. other cancer secretome datasets. (**A**) Breast cancer. (**B**) Comparative cancer datasets.

A comparative analysis on BC secretome and available secretomic data of other malignancies was performed to identify clinically relevant diagnostic and prognostic biomarkers applicable to BC and other adenocarcinomas. A total of 33 studies of breast cancer and 78 of other malignancies (comparative secretome) were screened for eligibility (Figure 1). Following eligibility screening according to inclusion and exclusion criteria, a total of 26 datasets remained. Eight (N = 8) breast cancer secretome datasets [38,39,40,41,42,43,44,45] and eighteen (N = 18) datasets of other cancers [25,26,46,47,48,49,50,51,52,53,54,55,56,57,58,59,60,61] were included in the comparative analysis, namely, colorectal (N = 2), gastric (N = 1), hepatocellular (N = 1), melanoma (N = 3), non-small cell lung adenocarcinoma (N = 2), ovarian (N = 4), pancreatic (N = 4), and prostate cancer (N = 1) (Tables S1 and S2). Most studies employed cell lines for secretome analysis and subtypes, including grade, and were as stated in Table S3. Three studies, one exploring BC, one colorectal, and one gastric cancer secretome, employed patient samples [39,46,48]. Datasets were normalized, and only proteins present in four (present in 50%) or more of included BC datasets were incorporated in the analysis. Regarding the BC secretome, we categorized cell strains and patient samples (Figure S1, Table S3) according to grade and merged into clusters. Cluster 1 corresponded to experiments of grade I and II malignancies while grade III malignancies were categorized into cluster 2.

### 3.2. BC Secretome Dataset Reconstruction and Mining

**Figure 2:**
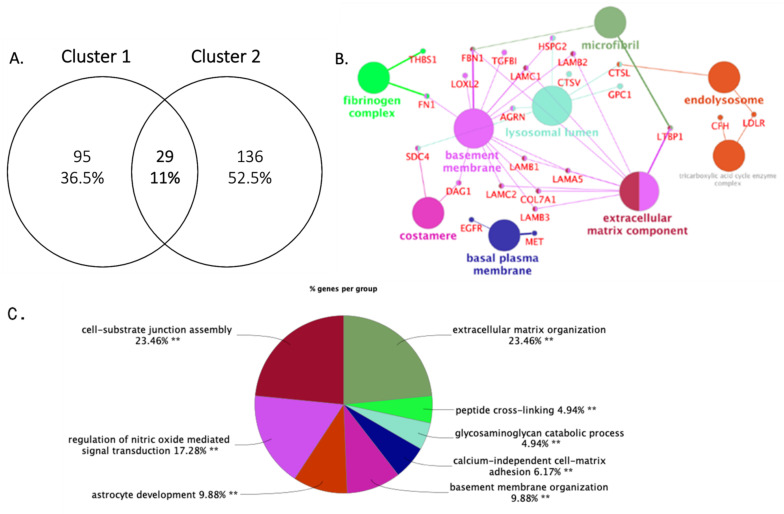
(**A**) Venn diagram of secretome overlap between grade I and II vs. grade III BC secretome. Overlap proteins’ cellular compartment localization (**B**), and biological process analysis (**C**). The network was constructed employing ClueGo V 2.3.2 plugin of Cytoscape V3.5.1. All displayed biological processes were enriched with *p* > 0.05. Significance (*p* value); **: *p* ≤ 0.01. Term *p*-values displayed are corrected with Bonferroni-Holm step down.

Initially, we aimed to identify if a proportion of the secretome was shared between cluster 1 and 2 (Figure 2A). Differential levels of the shared proteins would potentially highlight core regulators of the transition between grade categorizations. A total of 11% (N = 29 out of 174) of the combined secretomic data was overlapping between cluster 1 and 2 (Figure 2A). In terms of Go_cellular compartment analysis, the majority were proteins that normally localize in the basement membrane and the extracellular matrix (Figure 2B). Enriched GO_Biological process analysis highlighted basement membrane organization (37.25%) and cell-substrate junction organization and assembly (29.41%, 13.73%, respectively), followed by ECM organization (5.88%), glycosaminoglycan catabolism (3.92%), and regulation of the transforming growth factor beta (TGFb) production (3.92%) (Figure 2C). Finally, enriched GO_Molecular function clusters included fibronectin (50%), virus receptor (16.67%), integrin (16.67%), and laminin-binding (16.67%).

**Figure 3:**
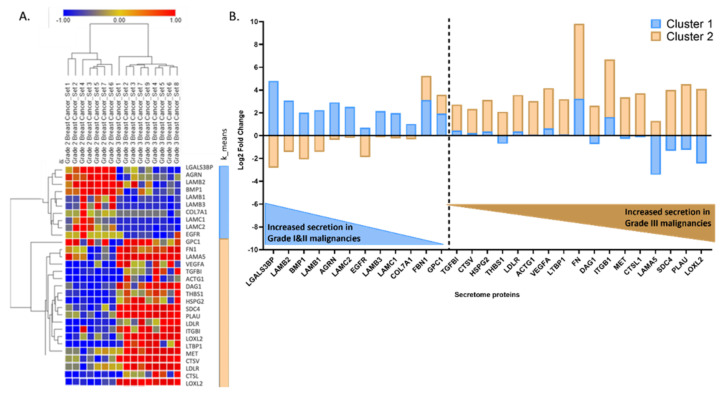
Common grade I and II and grade III BC secretome protein level heatmap (**A**) and prevalent proteins in grade I and II vs. grade III (**B**). Heatmap coloring; blue squares (−1-fold change); red [1-fold change (in comparison to wild type)]. Log2 fold change is measured as a log2 ratio of cancer cell line (mutant)/wild-type (control/reference) as reported in each study (**A**) Heatmap generated with Morpheus online freeware and (**B**) with GraphPad Prism V. 9.3.

Having identified this central cluster, we further sought to delineate whether protein levels within it differed between tumor grading. Two distinct groups were identified by hierarchical clustering of average log2 fold change across grade I and II vs. grade III datasets (Figure 3A). A total of 12 proteins were found to be more abundant in cluster 1 (grade I and II), namely, LGALS3BP, LAMB1, LAMB2, LAMB3, LAMC1, LAMC2, BMP1, AGRN, EGFR, COL7A1, FBN1, and GPC1, while 17 proteins, namely, TGFBI, CTSV, HSPG2, THBS1, LDLR, ACTG1, VEGFA, LTBP1, FN, DAG1, ITGB1, MET, CTSL1, LAMA5, SDC4, PLAU, and LOXL2, were found to be predominantly increased in cluster 2 (grade III) (Figure 3A,B).

### 3.3. BC and Other Adenocarcinoma Secretome Comparative Analysis

**Figure 4:**
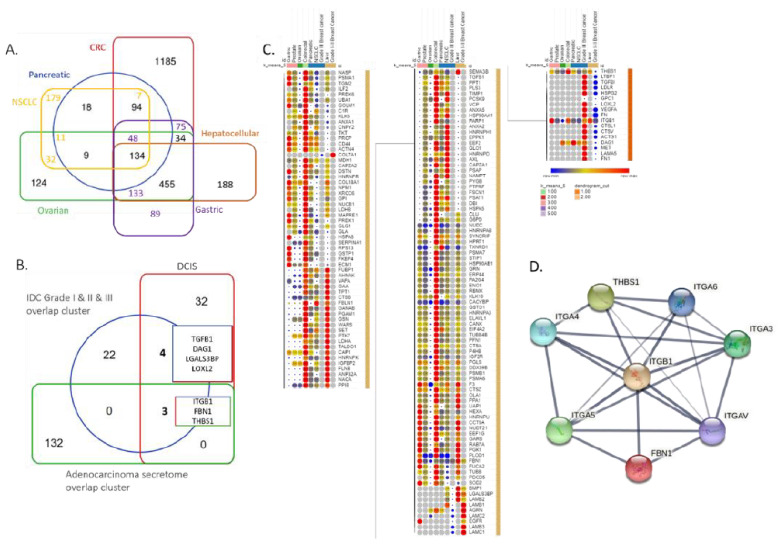
Venn diagram of overlap between comparative and breast cancer secretome (all grades). (**A**) Venn diagram to identify overlap amongst analyzed adenocarcinomas (pancreatic, ovarian, hepatocellular, and CRC). (**B**) Commonalities between central adenocarcinoma and BC clusters. (**C**) Heatmap of common clusters (adenocarcinomas, breast cancer). Red circles indicate upregulation, yellow indicate neither up- nor downregulation, whilst blue indicate downregulation of depicted proteins in comparison to the wild-type strain (reference) as reported in each original study. K-mean values and color coding depicted amongst cancer datasets. (**D**) DCIS biomarker list [62,63].

We further aimed to clarify whether these proteins were exclusive to BC secretome or could be widely identified in other malignancies. We systematically reviewed the literature to identify full secretomic datasets of other malignancies, namely, colorectal, gastric, hepatocellular, melanoma, non-small cell lung, ovarian, pancreatic, and prostate cancer. A total of 134 proteins were identified in all examined cancer secretomes, except for the melanoma dataset (Figure 4A). Intriguingly, metabolic processes, such as glycolysis/gluconeogenesis (15.4%) and pyruvate metabolism (15%), pentose phosphate pathway (7.7%), and glutathione metabolism (7.7%), were significantly enriched. This protein set was then cross compared with a central cluster of 29 proteins as identified between secretome comparison of grade I and II and III datasets as well as secretomic data of DCIS (Figure 4B) [62,63]. In the effort to recapitulate hallmarks of high-grade (HG) DCIS-IDC transition, we aimed to identify available DCIS secretomic data, where only a single dataset was identified in the literature. Of note, in the Mbeunkui et al. [62] study, only the highly abundant proteins in DCIS were reported, while the entirety of the dataset was not supplied, neither as a supplementary nor as a database registered set registration. Three proteins, namely, ITGB1, FBN1, and THBS1, were identified as common across the examined adenocarcinomas, DCIS and BC of all grades. A further four more proteins, namely, TGFβ1, DAG1, LGALSBP3, and LOXL2, were found to be common between DCIS and breast cancer (all grades) (Figure 4B). Hierarchical clustering was employed upon the common 29 proteins in BC and the 134 proteins overlapping across malignancies. The grade III BC dataset was more highly associated with pancreatic cancer and NSCLC, while the grade I and II BC dataset was more associated with hepatocellular carcinoma enriched proteins (Figure 4C). The ITGB1, FBN1, and THBS1 proteins were then analyzed to identify whether they participated in the same pathway, the nature of that pathway, and their associated first-degree interactors (Figure 4D). Enrichment of the KEGG pathways involving ECM receptor interaction and focal adhesion (FDR 1.36 × 10^−13^) as well as the PI3K-Akt signaling pathway (FDR: 2.39 × 10^−10^) was evident.

### 3.4. Central Cluster of BC Secreted Proteins and Patient Survival Correlations

**Figure 5:**
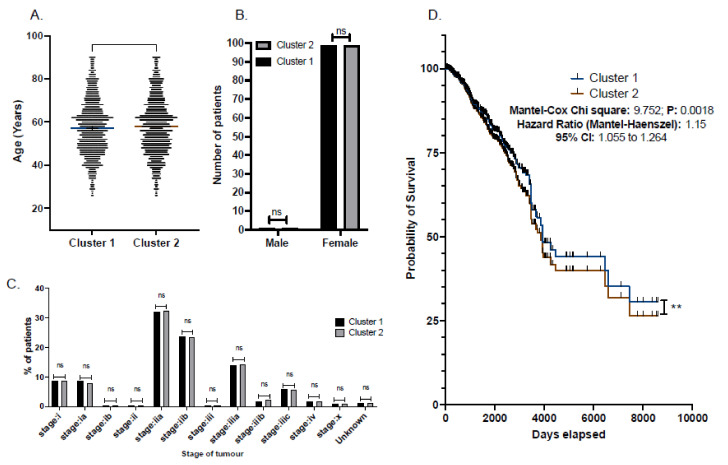
The expression of common grade I, II, and III BC secreted protein-encoding genes (above cut-off FPKM as per each individual gene transcript) predicted poor survival outcome across patients. Patient demographic (age and gender) (**A**,**B**) and disease staging (**C**) characteristics. Statistical significance assessed with unpaired *t*-test (**A**–**C**). Kaplan-Meier survival curve of mortality in breast cancer patients) were stratified into those with increased expression of proteins identified dominant in grade I and II (blue) vs. those with proteins identified dominant in grade III (brown) (**D**). Significance (*p* value); ns: *p* > 0.05; **: *p* ≤ 0.01. Survival curve, Mantel-Cox chi square and hazard ratio (Mantel-Haenzel) (95%CI) generated with GraphPad Prism V. 9.3.

Focusing on the 29 proteins identified amongst all grades of BC secretomic data (Figure 3), we further aimed to understand their implication on breast cancer patient mortality outcomes (Figure 5). We collected patient mortality data from the Human Protein Atlas, the increased abundance of cluster 1 or 2 proteins (Figure 3, Table S4). Summative mortality data were collected for 15,139 patients of various stages (File S1) with breast cancer (Figure 5). Females made up 98.93% of the entire patient population, while males made up 1.07%. The median age of the whole patient population was 58 years (range 26–90). Of note, a statistically significant difference (*p* = 0.012)) was observed between patients with cluster 1, where the median age was 57 years (range 26–90), in comparison to those with cluster 2 protein overexpression, where the median age was 58 years (range 26–90) (Figure 5A). No statistical difference was observed between cluster 1 to cluster 2 gender distribution (B) or tumor stage distribution (C). Survival curve analysis between patients overexpressing cluster 1 vs. patients overexpressing cluster 2 proteins, found that cluster 2overexpressing patients were at an increased risk of mortality, HR 1.15 (95% CI 1.05 to 1.26) (*p* = 0.015) (Figure 5D).

### 3.5. Pathway Reconstruction

**Figure 6:**
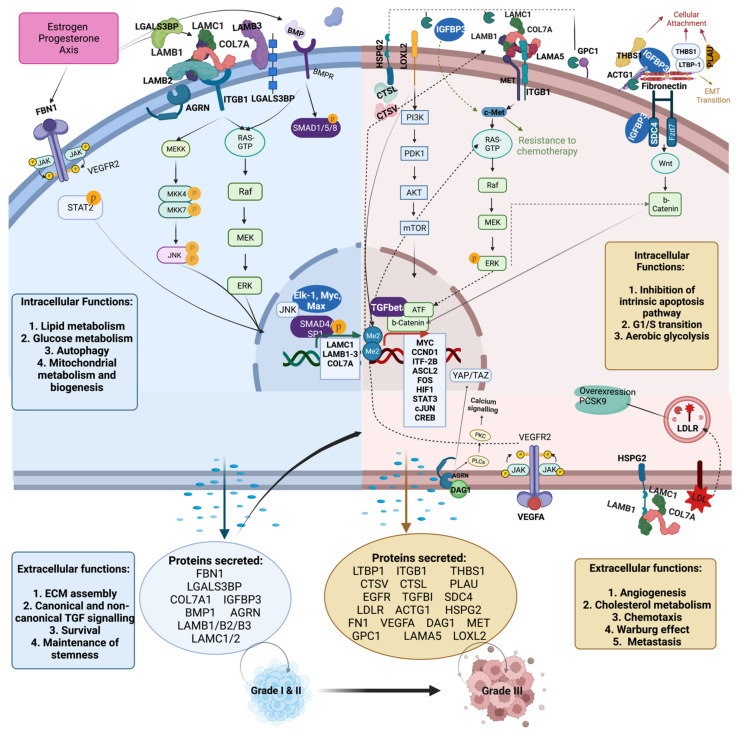
Proposed mechanism by which hierarchical secretion of identified secretome proteins may lead to the progression from grade I and II to grade III breast cancer Proteins in bold belong to the overlapping protein cluster as shown in Figure 3. Blue cell (grade I and II, Cluster 1) interactions: LAMB1-3, LAMC1, COL7A, and AGRN interactions with ITGB1 [65,66]. Downstream activation of JNK and ERK cascades through ITGB1 [67,68]. Parallel activation of the ERK cascade by EGF-EGFR interaction [69]. FBN1 interaction with VEGFR1 and downstream STAT2 activation [70]. BMP interaction with BMPR and subsequent activation of SMAD 1/5/8 [71]. Downstream effectors of JNK and ERK cascade [72]. Brown cell (grade III, cluster 2) interactions: HSPG2 and LOXL2 interaction with IGFB3 [38,73]. Laminin protein family interaction with MET and ITGB1 [74,75]. ACTG1 interaction with THBS1, LTBP1, fibronectin, and LOXL to drive SDC4 phosphorylation and subsequent WNT pathway activation [76,77,78,79,80]. LDLR laminin interaction and internalization leads to PCSK9 activation and tumor microenvironment promotion [81]. VEGFA-VEGFR2 interaction promotes the ERK cascade activation [82]. IGFBP-3, fibrillin, and ERK cascade leads activation of TGF-beta [83,84]. Integrin-beta1-dependent activation of MET [85]. mTOR downstream effectors [86]. TGFβ genetic targets [87]. Wnt and b-catenin downstream genetic targets [88]. Image created with BioRender.com. Protein name abbreviations stated in Table S6.

We further sought to reconstruct cluster 1 and cluster 2-enriched pathways to delineate whether cluster 1 and 2 proteins interact hierarchically in a temporal fashion to promote tumor upgrading (Figure 6). Protein—protein interactions of the 29 candidates constituting the overlapping cluster were analyzed through the STRING protein interaction database. Only physical subnetwork interactions identified experimentally or recorded in public databases, with a minimum requirement of a medium confidence [0.4] of interaction, were allowed to be displayed. A total of 71 edges (interactions) were generated amongst the input proteins (N: 29). Primary GO_KEGG pathways enriched were ECM-receptor interaction (FDR: 1.44 × 10^−19^), proteoglycans in cancer (FDR: 1.26 × 10^−15^), focal adhesion (FDR: 1.26 × 10^−15^), and PI3K-Akt signaling pathway (FDR: 1.26 × 10^−15^).

We then manually curated all displayed interactions to understand the interconnectivity of proteinic players and enriched pathways (Figure 6). In terms of cluster 1 proteins (grade I and II), LGALS3BP, LAMB1-3, LAMC1, COL7A, and AGRN form variable complexes, whilst the downstream effectors are extremely diverse, but for the purposes of this manuscript, we focused on the most extensively researched interaction, that with ITGB1, integrin beta-1, also known as CD29. ITGB1, through interaction with ITG alpha, forms the cellular collagen receptors. Activation of ITGB1 leads to activation of the JNK and ERK signaling pathway. In turn, that leads to JNK phosphorylation and transcription of downstream genetic targets as well as activation of Elk-1, Myc, and Max transcription factors and transcription of their dependent genetic targets. Other secreted proteins identified in cluster 1 included FBN1 and BMP, which through activation of VEGFR2 and BMPR lead to phosphorylation of STAT2 and SMAD 1/5/8 and respective nuclear translocation (Figure 6, blue cell). Activation of LAMC1, LAMB1-3, and COL7A encoding genes creates a positive feedback loop further escalating the expression of upstream receptors (Figure 6, blue cell). Overall, intracellular functions of the activated effectors lead to increase in lipid and glucose metabolism, mitochondrial biogenesis, and autophagy, whilst on the other hand, extracellular functions include ECM assembly, canonical and non-canonical TGF signaling, cellular survival, and maintenance of stemness. Of note, both ER-positive and triple-negative malignancies are driven by the estrogen and progesterone axis, albeit through different receptor activity (Figure 6, blue cell) [64].

In terms of proteins found more abundant in cluster 2 (grade III), LAMC1, LAMB1-3, and COL7A have been shown to interact with MET (MET proto-oncogene, receptor tyrosine kinase), and ITGB1, in turn activating the mTOR and ERK signaling cascades. Additionally, secreted fibronectin acts as a scaffold for ACTG1, THSB1, PLAU, and LTBP1 binding, which in turn leads to activation of the Wnt cascade through interaction with SDC4, Syndecan 4, a transmembrane (type I) heparan sulfate proteoglycan that functions as a receptor in intracellular signaling. Additionally, secretion of LOXL2, lysyl oxidase-like 2, and HSPG2, perlecan 1, in turn leading to mTOR signaling cascade and TGFb pathway activation. Additionally, DAG1 (dystroglycan 1) increased secretion leads to Hippo pathway and PKC cascade activation in response to hyperglycemia, whilst over-secretion and activation of LDLR leads to receptor internalization and subsequent PCSK9 overexpression and SRBP1, Insig-Srebp-Scap, pathway activation., which is a recognisedmetastasis driver in colorectal cancer. These cascades converge to drive expression of central players in apoptosis inhibition, resistance to chemotherapy phenotypes, and metastatic niche progression.

Overall, we suggest that preparatory synergism between the cascades identified as prominent in grade I and II BC samples act in a preparatory fashion to enable progression to grade III. Therefore, serological identification of LGALS3BP, LAMB1-3, LAMC2, BMP1, AGRN, EGFR, COL7A, FBN1, and GPC1 may prove of great clinical value in identifying metastatic potential of BC prior to pathological and imaging evidence. Equally, markers such as LGALSBP3 (predominant in grade I and II) and TGFΒ1, DAG1, and LOXL2 (predominant in grade I and II) may be able to provide further choice insights between operative and medical management of DCIS.

## 4. Discussion

Globally, breast cancer is the most frequently diagnosed cancer in women associated with a significant mortality. Secreted proteins constitute an important class of molecules, encoded by approximately 10% of the human genome, and inevitably their identification and exploitation use as biomarkers has been an attractive target for a variety of diseases, including cancer. Secretome integrative and comparative analysis in other malignancies such as pancreatic and lung cancer has offered multiple putative biomarkers of cancer diagnosis and progression, some of which have clinical applicability [89,90,91].

Here, we have presented the first BC secretome integrative and comparatice analysis and identified putative predictors of DCIS to IDC (TGFβ1, DAG1, LGALSBP3, and LOXL2) as well as grade I and II IDC to grade III IDC (LGALS3BP, LAMB1-3, LAMC2, BMP1, AGRN, EGFR, COL7A, FBN1, and GPC1) progression. By utilizing in silico pathway prediction, we also reconstructed an integrative pathway through which the grade I and II to III transition may be mediated. We also identified biomarkers, namely, ITGB1, FBN1, and THBS1, secreted by BC cells constituting lesions of all grades as well as the examined adenocarcinomas (colorectal, gastric, hepatocellular, melanoma, non-small cell lung cancer, ovarian, pancreatic, and prostate cancer). These biomarkers may have clinical applicability in terms of non-invasive and non-radiation-based screening. Lastly, we correlated the presence of grade III BC secreted proteins with poorer survival outcomes in comparison to those of grade I and II in breast cancer patients.

The identification of the above-mentioned biomarkers may raise a slew of clinical questions. Firstly, whether experimentally validated data exist that imply the value of TGFB1, DAG1, LGALSBP,3 and LOXL2 as DCIS to IDC progression predictors (Figure S2, Table S5). Recently a study by Evans et al. [92] showed that TGFB1 mRNA levels have been associated with increased risk of DCIS to IDC transition with HR of 7.85 (95% CI 1.92–32.11). Single-cell RNA sequencing has also highlighted the implications of LGALSBP3 upregulation upon poorer patient outcomes [93]. DAG1 function in breast cancer has been suggested to be of paracrine nature through hAG/DAG-1 interaction and thus involved in tumor microenvironment organization [94]. Lastly, LOXL2 secretion has been shown to induce collagen crosslinking and adaptation via acidosis, promoting survival and growth of nascent cancers [95]. Taken into the context of the present study that highlights the abundance of these proteins in the secretome of DCIS, it can be hypothesized that immunohistochemical or aspirate detection of these proteins may provide insight of the tumor microenvironment and therefore potentially be studied as potential predictive tools to inform clinical decision making in the context of DCIS management in a non-invasive manner.

Another question of clinical relevance that arises regarding the herein presented data is whether secretome proteins such as HSPG2 [96], MET [97,98], SDC4 [99,100], ACTG1 [101,102], VEGFA [103], FN1 [104], CTSL1 [105,106], and LAMA5 [107] could be used as biochemical transition markers between grades. An important discriminator of such a biomarker would be its increasing serological abundance in malignant states but not in other inflammatory or infectious pathologies. As such, from the above-mentioned biomarkers, while all have been extensively associated with cancer states, only HSPG2 [96], ACTG1 [101,102], and LAMA5 [107] are not significantly elevated in other non-cancerous conditions, e.g., infection, auto-immunity, and ischemia (Figure S2, Table S5). Intriguingly, HSPG2 has been recently identified as a promising target in both metastatic ER positive and TNBC with HSPG2-targeted antibodies being suggested as a potentially novel class of targeted therapeutics for TNBC [96,108].

Finally, could serological detection of proteins such as ITGB1, FBN1, and THBS1 be employed as a general screening tool for adenocarcinomas (Figure S2, Table S5)? Over-expression of these proteins has been extensively implicated in a variety of malignancies’ progression and metastasis. ITGB1, FBN1, and THBS1 have not been coherently identified as putative “pan-cancer” early detection biomarkers because a similar study to this one has not been conducted previously [109,110,111,112,113,114]. Nonetheless, the notion of other pancancer biomarkers in prognostic detection has already been tested with promising results as highlighted in the DETECT-A clinical trial [115,116]. Intriguingly, chemical inhibitor of ITGB1 is under development (Patent PubChem number: CN-113198017-A).

Whilst proteinic markers have been extensively analyzed in the context of disease identification and prognosis, a special mention is required considering the increasing clinical momentum of liquid biopsy, e.g., identification of circulating free tumor DNA (cfDNA) in patient biological fluids, in breast cancer diagnostics [117]. Despite the fact that the sensitivity of conventional next-generation sequencing (NGS) in detecting DNA alterations is finite as it requires a high fraction of cancerous to wild-type DNA copies [118], recent studies have developed a targeted error correction sequencing (TEC-Seq) approach to detect cfDNA sequence changes through a highly-sensitive massive genome sequencing pipeline analyzing oncogenic genes in a multitude of malignancy types, namely, early-stage colorectal, breast, lung, and ovarian cancer in a sample of 200 patients [119]. Somatic mutations were detected in 71%, 59%, 59%, and 68% of cases, respectively. In fact, the utility of combined liquid biopsy cfDNA and protein biomarkers in increasing detection specificity has also been recently highlighted by the CancerSEEK multi-analyte blood test [120]. CancerSEEK evaluated both cfDNA mutations in 1.005 clinically diagnosed stage I-III cancer patients, including early BC as well as circulating levels of eight serum protein biomarkers significantly improving detection sensitivity. Therefore, a combinatorial approach may significantly benefit malignancy detection especially in low cancerous to wild-type DNA alterations.

### Strengths and Limitations

The present hypothesis-generating in silico study represents the first breast cancer secretome systematic review of literature integrative and comparative analysis versus other adenocarcinoma datasets. Our study highlights novel putative biomarkers that may underpin DCIS transition to IDC but enable IDC grade progression. Nonetheless, it should be noted that only a single study offered DCIS secretomic data, and therefore study evidence should be interpreted with caution. Furthermore, pathway prediction analysis and patient survival data have been integrated to provide both a molecular and clinical basis of the putative function of the suggested biomarkers in the context of malignancy detection, prognosis, and metastatic potential. Albeit only sufficiently homogeneous secretomic datasets were pooled to generate the integrated secretomic data presented here, with variability stemming from the different setting of original data acquisition, including laboratory environment, quantification approach, and human factors potentially inevitably having introduced batch effects. Equally, it has been previously demonstrated that even different batches of the same cell line may harbor a degree of genetic heterogeneity, which in turn can lead to variations in protein expression [120,121]. Of note, cell lines do display overall similar levels of variability in protein expression when compared to whole tissue samples, but nonetheless expression of molecules, such as cell—cell adhesion proteins and receptors, have been shown to significantly vary between cell line and tissue samples [122]. Additionally, the tumor-grade-related biomarkers highlighted in the present study were primarily identified through comparative analysis of cell lines rather than patient tumor samples. Therefore, robust, comparative tumor sample data validation is of paramount importance prior to any further biomarker clinical evaluation. Lastly, regarding the survival data analysis of breast cancer patients with increased expression of cluster 1 or 2 proteins presented herein, it should be noted that whilst patient age, ethnicity, and tumor stage were available, tumor grade, however, was not, and therefore could not be adjusted for. This presents another inherent limitation of the publicly available datasets (Human Protein Atlas), which in turn may have skewed the survival analysis curve and subsequently its statistical significance.

To minimize such variability, a pooled, integrative approach was employed to resynthesize data. The strength of such an integrative bioinformatic approach relies on the minimization of single experiment variations and biases that are introduced by specific cell lines and malignancy hormonal receptor status and human- and laboratory-specific factors. To guard against bias in the undertaking of the review, two reviewers independently extracted all data, and where disagreement occurred, this was discussed between authors to reach a consensus. Nonetheless, whilst available literature evidence uniformly suggests that the herein identified biomarkers may have significant clinical implications, the corroboration with targeted clinical data and robust randomized clinical trials remain to be actioned. Validation of these targets should be the focus of future work.

## 5. Conclusions

The present study highlights an abundance of putative secretome biomarkers that may provide insight of the tumor microenvironment and therefore inform clinical decision making in the context of IDC management in a non-invasive manner.

## Data Availability

The data presented in this study are available in the Supplementary Materials.

## Supplementary Materials

The following supporting information can be downloaded at https://www.mdpi.com/article/10.3390/cancers14163854/s1, Figure S1: Identification of common proteins across BC secretome datasets: (A) tumors categorized as grade I and II and (B) tumors categorized as grade III. Figure S2: Blood levels of selected biomarkers (micrograms/Litre). DCIS to IDC progression biomarkers: TGFB1, DAG1, LGALS3BP, and LOXL2 (teal). IDC grade progression: HSPG2, ACTG1, and LAMA5 (plum). Pan-cancer biomarkers: ITGB1, FBN1, and THBS1 (cayenne). Table S1: Datasets included in the breast cancer secretome comparative analysis. Table S2: Datasets included in other cancer secretome comparative analysis. Table S3: Cell lines used in breast cancer and other malignancy integrative and comparative analysis. (accessed on 26 July 2022) Table S4: Human Protein Atlas prognosis and biological process of BC and comparative cancer patients for proteins identified in grade I and II BC vs. grade III BC clusters. Table S5. Blood levels of selected biomarkers. Table S6: Abbreviation list.
