# Supplementary material for: Integrative, In Silico and Comparative Analysis of Breast Cancer Secretome Highlights Invasive-Ductal-Carcinoma-Grade Progression Biomarkers
Source: Cancers (Basel). 2022 Aug 9;14(16):3854. doi: 10.3390/cancers14163854 (PMC9406168; doi:10.3390/cancers14163854)
Supplement: Supplementary file 1 [file cancers-14-03854-s001.zip › cancers-1828998-supplementary.pdf]

Integrative, In Silico and Comparative Analysis of Breast Cancer Secretome Highlights Inva-sive-Ductal-Carcinoma-Grade Progression Biomarkers

Stavroula L. Kastora [1,2]*, Georgios Kounidas [1], Valerie Speirs [1] and Yazan A. Masannat [1,2]

Supplementary Materials

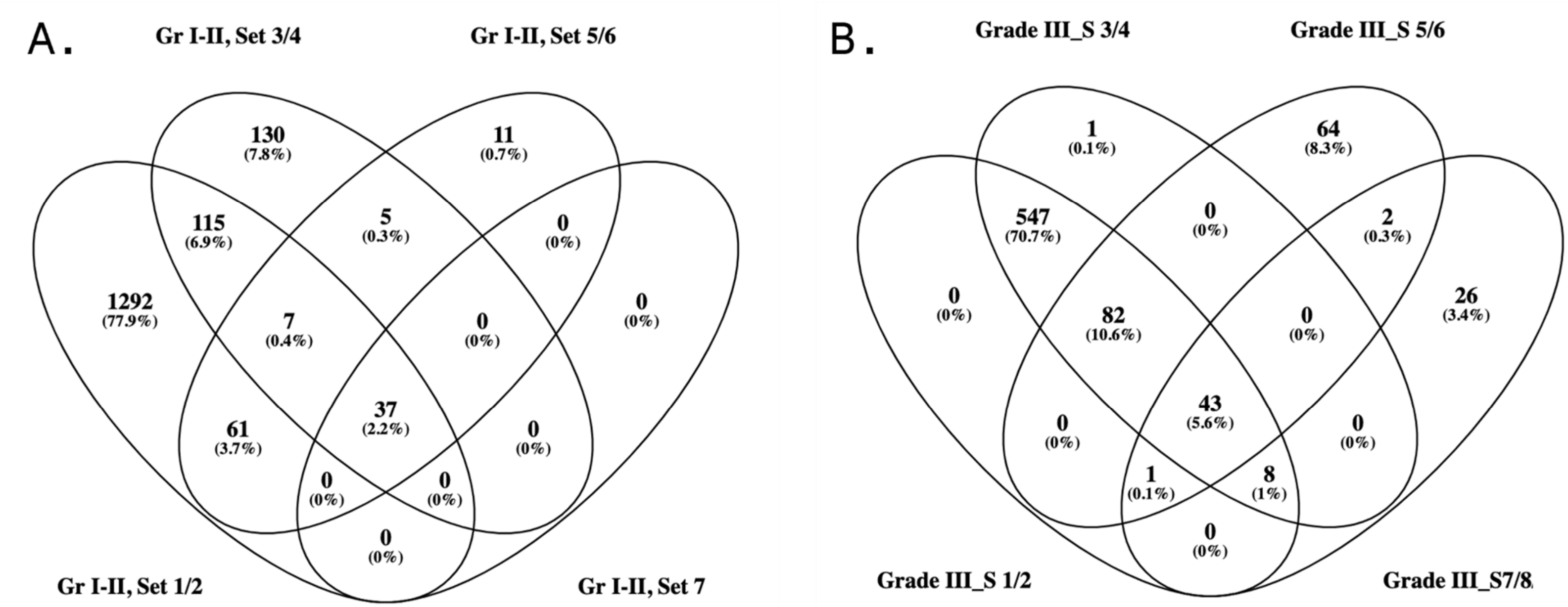

Figure S1. Identification of common proteins across BC secretome datasets (**A**) Tumours categorised as Grade I & II and (**B**) Tumours categorised as Grade III.

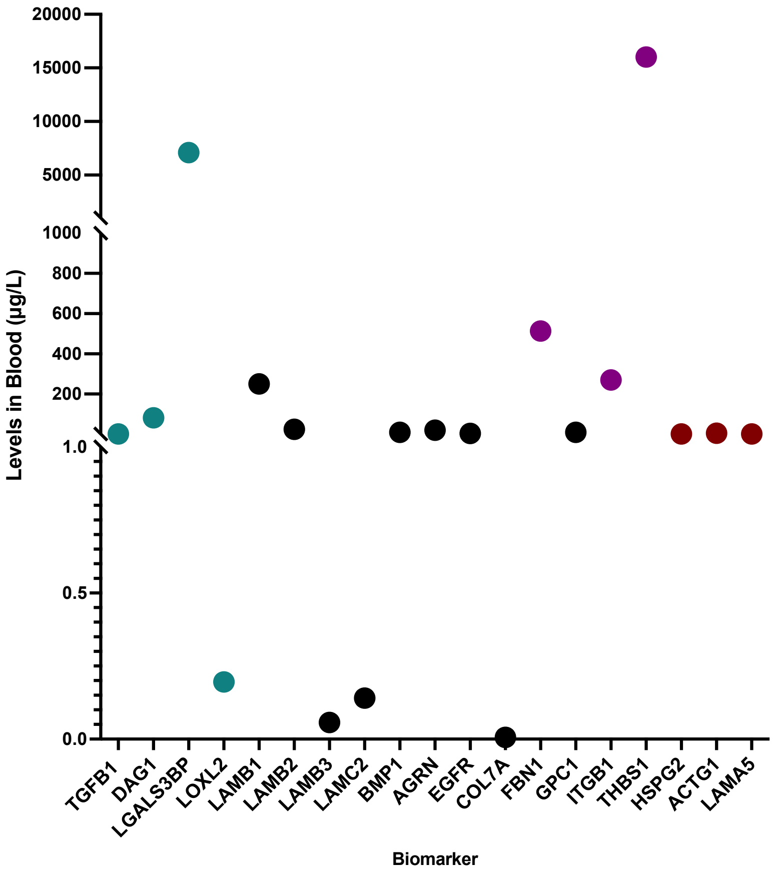

Figure S2. Blood levels of selected biomarkers (micrograms/Litre). DCIS to IDC progression biomarkers; TGFB1, DAG1, LGALS3BP, LOXL2 (Teal). IDC grade progression; HSPG2, ACTG1 and LAMA5 (Plum), Pan-cancer biomarkers; ITGB1, FBN1 and THBS1 (Cayenne).

Table S1. Datasets included in the breast cancer secretome comparative analysis.

Title	Author	ProtCode	Cancer	Cell line	Equipment
Integrative analysis of mesenchymal stromal cell conversion by breast cancer secretomes in blank slate 3D microenvironments	Blache et al.	PXD010467	Breast cancer	MCF-7, MDA-MB-231	IC-MS
Differential proteomic comparison of the breast cancer secretome using a quantitative paired analysis workflow	Brunoro et al.	PXD005157	Breast cancer	**Patient samples (ER positive)**	IC-MS; LIT-MS
Breast Cancer Metastasis Organotropism	Zhuang et al.	PXD007227	Breast cancer	MDA-MB-231	LIT-MS
MDA-MB-231 Breast Cancer Bone Tropic vs Parental Hypoxic Secretome Analysis	Cox et al.	PXD000397	Breast cancer	MDA-MB-231	IC-MS
Novel secretome-to-transcriptome integrated or secreto-transcriptomic approach to reveal liquid biopsy biomarkers for predicting individualized prognosis of breast cancer patients.	Ankney et al.	-	Breast cancer	MCF10A, MCF7, MDA-231, T47D, and HCC1806	Tribrid-MS

Quantification of the N-glycosylated secretome by super-SILAC during breast cancer progression and in human blood Samples.	Boersema et al.	-	Breast cancer	HMT-3522-S1; MFM223; HCC202, HCC2218; HCC1143, HCC1937, HCC1599; MCF-10a and MDA-MB-453	SILAC
Differential secretome analysis reveals CST6 as a suppressor of breast cancer bone metastasis.	Jin et al.,	-	Breast cancer	MCF10; MDA231	IC-MS
Spontaneous regression of micro-metastases following primary tumor excision: a critical role for primary tumor secretome	Shaashua et al.	PXD008384	Breast cancer	MDA-MB-231	IC-MS

IC-MS Ion Chromatography Mass Spectrometry; LIT-MS linear ion trap Mass Spectrometry; SILAC Stable isotope labeling by amino acid in cell culture; Tribrid-MS: quadrupole, ion trap and Orbitrap mass analysis

Table S2. Datasets included in other cancer secretome comparative analysis.

**Title**	**Author**	ProtCode	**Cancer origin**	**Cell line**	**Equipment**
Multi-layered proteomic analysis for colorectal cancer	Kwon et al.	PXD018304	Colorectal cancer	**Patient samples**	IC-MS; LIT-MS
In-depth characterization of the secretome of colorectal cancer metastatic cells identifies key proteins in cell adhesion, migration, and invasion.	Barderas et al.	PXD030671	Colorectal cancer	KM12C KM12SM; KM12L4a SW480; SW620	Q Exactive HF
Characterization of phosphoproteins in gastric cancer secretome.	Yan et al.	PXD002674	Gastric cancer	**Patient samples** (ERBB2 positive & negative)	LIT-MS
Identification of CHI3L1 and MASP2 as a biomarker pair for liver cancer through integrative secretome and transcriptome analysis.	Wang J et al.	PXD025623	Hepatocellular carcinoma	Huh-7	LC-MS
PPARgamma regulates a cell-to-cell communication program in human methastatic melanoma cells	Pich et al.	PXD009108	Melanoma	A375, SkMel28	Tribrid-MS; LC-MS
Human melanoma cell lines secretome analysis	Liberato et al.	PXD007265	Melanoma	Hs 895.T (CRL-7636™), Hs 895.Sk (CRL-7637™), A375, (CRL-1619™), SH-4 (CRL-7724)	LIT-MS

Secretome analysis of human A549 Lung cancer cells harboring LKB1 mutations.	Granado-Martinez et al.	PXD018041	Lung cancer **Melanoma**	A549 lung cancer cells, G361 melanoma (Raw data available only for melanoma	LIT-MS
Proteome Analysis of Non-Small Cell Lung Cancer Cell Line Secretomes and Patient Sputum Reveals Biofluid Biomarker Candidates for Cisplatin Response Prediction	Bottger et al.	PXD011315	NSCLC	W1573, H2228, H522, H1650, H1975 and H1703	LIT-MS; LC-MS
Importin subunit alpha-2 is identified as a potential biomarker for non-small cell lung cancer by integration of the cancer cell secretome and tissue transcriptome.	Wang et al.,	PXD000861	NSCLC	PC9	LIT-MS
Cytokine-induced GBP1 release from human ovarian cancer cells	Carbotti et al.	PXD016479	Ovarian cancer	SKOV3 , OVCAR5, IGROV1, IST-A161	Tribrid-MS
Ovarian high grade serous carcinoma ascites cell secretome	Worzfeld et al.	PXD008047	Ovarian cancer	**Patient samples with HGSOC**	LC-MS
Analysis of ovarian cancer cell secretome during epithelial to mesenchymal transition reveals a protein signature associated with advanced stages of ovarian tumors.	Lanfredi et al.	PXD022040	Ovarian cancer	Caov-3	LC-MS
Integration of Two In-depth Quantitative Proteomics Approaches Determines the Kallikrein-related	Silva et al.	PXD003752	Ovarian cancer	OVMZ-6, SKOV-3	SILAC

Peptidase 7 (KLK7) Degradome in Ovarian Cancer Cell Secretome.					
MYB-regulated pancreatic cancer secretome	Zubair et al.	PXD015126	Pancreatic cancer	BxPC3-MYB	LIT-MS
Secretome protein signature of human pancreatic cancer stem-like cells.	Brandi et al.	Full dataset available online S. material	Pancreatic cancer	Panc1	LC-MS
Secretome-based identification of ULBP2 as a novel serum marker for pancreatic cancer detection.	Chang et al.	Full dataset available online S. material	Pancreatic cancer	BxPC-3, MIA PaCa-2, Panc1, AsPC-1	LC-MS
Characterization of soluble and exosomal forms of the EGFR released from pancreatic cancer cells.	Adamczyk et al.,	Full dataset available online S. material	Pancreatic cancer	BxPC3, MiaPaca2, Panc1	ESI-MS
Proteomics approach to identify novel metastatic bone markers from the secretome of PC-3 prostate cancer cells.	Sung et al.,	Full dataset available online S. material	Prostate cancer	PC-3	LC-MS

IC-MS: Ion Chromatography Mass Spectrometry; LIT-MS: linear ion trap Mass Spectrometry; SILAC: Stable isotope labelling by amino acid in cell culture; Tribrid-MS: quadrupole, ion trap and Orbitrap mass analysis; ESI-MS: **electrospray ionization mass spectrometry**

Table S3. Cell lines used in breast cancer and other malignancy integrative and comparative analysis. (accessed on the 26th of July, 2022)

**Cell strains**	
MCF10A	Non-malignant breast cell line; benign fibrocystic disease
MDA-231	Invasive ductal carcinoma Grade II
MCF7	Invasive ductal carcinoma Grade III
T47D	Invasive ductal carcinoma Grade II
HMT-3522-S1	Benign breast tumour
MFM223	Invasive ductal carcinoma, Grade III
HCC202	Invasive ductal carcinoma, Grade III, ER-, PR-
HCC2218	Invasive ductal carcinoma, Grade III, ER-, HER2+
HCC1143	Invasive ductal carcinoma, Grade III, Triple -
HCC1937	Invasive ductal carcinoma, Grade III, BRCA1 mutant, Triple negative
HCC1599	Invasive ductal carcinoma, Grade III, Triple negative
MDA-MB-453	Invasive ductal carcinoma Grade III
PC-3	Metastatic Grade IV prostate cancer
MiaPaca2	Invasive Pancreatic adenocarcinoma, Grade not stated
Panc1	Invasive Pancreatic epithelioid Carcinoma, Grade not stated
AsPC-1	Invasive Pancreatic adenocarcinoma, Grade not stated
MIA PaCa-2	Invasive Pancreatic epithelioid Carcinoma, Grade not stated
BxPC3-MYB	Invasive Pancreatic adenocarcinoma, Grade not stated
OVMZ-6	Invasive ovarian serous cystadenocarcinoma, Grade IV
SKOV-3	Invasive ovarian adenocarcinoma, Grade IV
Caov-3	High grade ovarian serous adenocarcinoma, Grade III
OVCAR5	High grade ovarian serous adenocarcinoma, Grade IV
IGROV1	Ovarian endometrioid adenocarcinoma, Grade not stated
IST-A161	Pleural epithelioid mesothelioma, Grade not stated
PC9	Lung adenocarcinoma, Grade not stated

H1703	Lung squamous cell carcinoma, Grade I
W1573	Lung adenocarcinoma, Grade I
H2228	Non-small cell lung adenocarcinoma, Grade not stated
H522	Non-small cell lung adenocarcinoma, Stage 2
H1650	Bronchoalveolar lung adenocarcinoma, Stage 3B
H1975	Non-small cell lung adenocarcinoma, Grade not stated
H1703	Non-small cell lung adenocarcinoma, Grade not stated
Huh-7	Hepatocellular adenocarcinoma, Grade not stated
G361	Melanoma, Grade not stated
Hs 895.T (CRL-7636™)	Melanoma, Grade not stated
Hs 895.Sk (CRL-7637™)	Benign, Fibroblast control
A375 (CRL-1619™)	Melanoma, Grade not stated
SH-4 (CRL-7724)	Melanoma, Grade not stated
SkMel28	Melanoma, Grade not stated
KM12C	Colon adenocarcinoma, Grade not stated
KM12SM	Colon adenocarcinoma, Grade not stated
KM12L4a	Colon adenocarcinoma, Grade not stated
SW480	Colon adenocarcinoma, Grade not stated
SW620	Colon adenocarcinoma, Grade not stated

ER: Estrogen receptor; PR: Progesterone receptor; HER2: Receptor tyrosine-protein kinase erbB-2, Neu/ERBB2.

Table S4. Human Protein Atlas prognosis and biological process of BC and comparative cancer patients for proteins identified in grade I and II BC vs. grade III BC clusters.

	**Protein**	**Name**	**Biological Process (UniProt)**	**Prognosis**	**Specificity**	**Predicted location**	**Detected in Secretome Dataset**	**Novel secreted function ?**
Grade I-II increased	LGALS3BP	Galectin 3 binding protein	Cell adhesion	Unfavourable	Low_All	Secreted	Secreted	No
Grade I-II increased	LAMB2	Laminin subunit beta 2	Cell adhesion	Unknown	Low_All	Membrane, Secreted	Secreted	No
Grade I-II increased	BMP1	Bone morphogenetic protein 1	Chondrogenesis, Differentiation, Osteogenesis	Prognostic marker in renal cancer (unfavourable)	Low_All	Secreted	Secreted	No
Grade I-II increased	LAMB1	Laminin subunit beta 1	Cell adhesion	Unfavourable	Low_All	Intracellular, Secreted	Secreted	No
Grade I-II increased	AGRN	Agrin	Differentiation	Unfavourable	Low_All	Intracellular, Secreted	Secreted	No
Grade I-II increased	LAMC2	Laminin subunit gamma 2	Cell adhesion	Unfavourable	Medium_Many_Specific Head and Neck	Secreted	Secreted	No
Grade I-II increased	EGFR	Epidermal growth factor receptor	Host-virus interaction	Unfavourable	Medium_Many_Specific Glioma	Intracellular, Membrane, Secreted	Secreted	No
Grade I-II increased	LAMB3	Laminin subunit beta 3	Cell adhesion	Prognostic marker in renal cancer (unfavourable), lung cancer (unfavourable),	Medium_Many_Specific	Intracellular, Secreted	Secreted	No

				pancreatic cancer (unfavourable) and breast cancer (favorable)	Head and Neck			
Grade I-II increased	LAMC1	Laminin subunit gamma 1	Cell adhesion	Unfavourable	Low_All	Intracellular, Secreted	Secreted	No
Grade I-II increased	COL7A1	Collagen type VII alpha 1 chain	Cell adhesion	Prognostic marker in pancreatic cancer (unfavourable) and head and neck cancer (favorable)	Medium_Many_Specific Head and Neck	Intracellular, Secreted	Secreted	No
Grade I-II increased	GPC1	Glypican 1	-	Prognostic marker in liver cancer (unfavourable), colorectal cancer (unfavourable) and endometrial cancer (unfavourable)	Low_All	Intracellular, Membrane, Secreted	Secreted	Yes
Grade III increased	TGFBI	Transforming growth factor beta induced	Cell adhesion, Sensory transduction, Vision	Prognostic marker in renal cancer (unfavourable), cervical cancer (unfavourable), ovarian cancer (unfavourable) and endometrial cancer (favorable)	Low_All	Intracellular, Secreted	Secreted	No
Grade III increased	CTSV	Cathepsin V	Angiogenesis	Prognostic marker in liver cancer (unfavourable), urothelial cancer (unfavourable), pancreatic cancer (unfavourable) and endometrial cancer (favorable)	Low_All	Intracellular, Secreted	Secreted	No

Grade III increased	HSPG2	Heparan sulfate proteoglycan 2	-	Prognostic marker in lurothelial cancer (unfavourable),	Low_All	Secreted to ECM	Secreted	No
Grade III increased	THSB1	Thrombospondin 1	Cell adhesion, Unfolded protein response	Prognostic marker in renal cancer (unfavourable) and stomach cancer (unfavourable)	Low_All	Secreted	Secreted	No
Grade III increased	LDLR2	Low density lipoprotein receptor	Cholesterol metabolism, Endocytosis, Host-virus interaction, Lipid metabolism, Lipid transport, Steroid metabolism, Sterol metabolism, Transport	Prognostic marker in pancreatic cancer (unfavourable), urothelial cancer (unfavourable) and renal cancer (unfavourable)	Low_All	Intracellular, Membrane Secreted	Secreted	No
Grade III increased	ACTG1	Actin gamma 1	-	Prognostic marker in colorectal cancer (favourable)	Low_All	Intracellular, Secreted	Secreted	No
Grade III increased	VEGFA	Vascular endothelial growth factor A	Angiogenesis, Differentiation	Prognostic marker in renal cancer (unfavourable), liver cancer (unfavourable), endometrial cancer (unfavourable), urothelial cancer (favorable) and	Low_All	Intracellular, Secreted	Secreted	No

				cervical cancer (unfavourable)				
Grade III increased	LTBP1	Latent transforming growth factor beta binding protein 1	-	Prognostic marker in renal cancer (unfavourable) and urothelial cancer (unfavourable)	Low_All	Intracellular, Secreted	Secreted	No
Common in all adeno+BC (more in Grade I/II)	FBN1	Fibrillin 1	-	Prognostic marker in renal cancer (unfavourable), urothelial cancer (unfavourable) and stomach cancer (unfavourable)	Low_All	Secreted	Secreted	No
Grade III increased	DAG1	Dystroglycan 1	Host-virus interaction	Prognostic marker in renal cancer (favorable)	Low_All	Intracellular, Membrane Secreted	Secreted	No
Common in all adeno+BC (more in Grade III)	ITGB1	Integrin subunit beta 1	Cell adhesion, Host-virus interaction	Unfavourable	Low_All	Intracellular, Membrane, Secreted	Secreted	No
Grade III increased	MET	MET proto-oncogene, receptor tyrosine kinase	-	Prognostic marker in pancreatic cancer (unfavourable) and head and neck cancer (unfavourable)	Low_All	Intracellular, Membrane, Secreted	Secreted	No
Grade III increased	CTCL	TSPY like 2	Cell cycle, Transcription, Transcription regulation	Prognostic marker in pancreatic cancer (favorable), renal cancer (unfavourable) and colorectal cancer (unfavourable)	Low_All	Intracellular	Secreted	Yes

Grade III increased	LAMA5	Laminin subunit alpha 5	-	Unknown	Unknown	Intracellular, Secreted	Secreted	No
Grade III increased	FN1	Fibronectin 1	Acute phase, Angiogenesis, Cell adhesion, Cell shape	Prognostic marker in renal cancer (unfavourable), stomach cancer (unfavourable) and urothelial cancer (unfavourable)	Medium_Many_Specific thyroid cancer	Intracellular, Secreted	Secreted	No
Grade III increased	SDC4	Syndecan 4	-	Prognostic marker in renal cancer (favorable), thyroid cancer (favorable) and pancreatic cancer (unfavourable)	Low_All	Membrane, Secreted	Secreted	No
Grade III increased	PLAU	Plasminogen activator, urokinase	Blood coagulation, Fibrinolysis, Hemostasis, Plasminogen activation	Prognostic marker in pancreatic cancer (unfavourable), head and neck cancer (unfavourable), endometrial cancer (favorable), renal cancer (unfavourable) and lung cancer (unfavourable)	Low_All	Intracellular, Secreted	Secreted	No
Grade III increased	LOXL2	Lysyl oxidase like 2	Transcription, Transcription regulation	Prognostic marker in lung cancer (unfavourable), renal cancer (unfavourable), cervical cancer (unfavourable), glioma (unfavourable) and pancreatic cancer (unfavourable)	Low_All	Intracellular, Secreted	Secreted	No

Table S5. Blood levels of selected biomarkers.

initial_alias	converted_alias	description	Confirmed in Human Protein Atlas	Blood concentration (micrograms/L)
TGFB1	ENSG00000105329	transforming growth factor beta 1 [Source:HGNC Symbol;Acc:HGNC:11766]	Secreted	1.3
DAG1	ENSG00000173402	dystroglycan 1 [Source:HGNC Symbol;Acc:HGNC:2666]	Secreted	84
LGALS3BP	ENSG00000108679	Galectin 3 binding protein	Secreted	7100
LOXL2	ENSG00000134013	lysyl oxidase like 2 [Source:HGNC Symbol;Acc:HGNC:6666]	Secreted	0.193
LAMB1	ENSG00000091136	laminin subunit beta 1 [Source:HGNC Symbol;Acc:HGNC:6486]	Secreted	250
LAMB2	ENSG00000172037	laminin subunit beta 2 [Source:HGNC Symbol;Acc:HGNC:6487]	Secreted	25
LAMB3	ENSG00000196878	laminin subunit beta 3 [Source:HGNC Symbol;Acc:HGNC:6490]	Secreted	0.02
LAMC2	ENSG00000058085	laminin subunit gamma 2 [Source:HGNC Symbol;Acc:HGNC:6493]	Secreted	0.13
BMP1	ENSG00000168487	bone morphogenetic protein 1 [Source:HGNC Symbol;Acc:HGNC:1067]	Secreted	9.2
AGRN	ENSG00000188157	agrin [Source:HGNC Symbol;Acc:HGNC:329]	Secreted	20
EGFR	ENSG00000146648	epidermal growth factor receptor [Source:HGNC Symbol;Acc:HGNC:3236]	Secreted	4.7
COL7A	ENSG00000114270	Collagen type VII alpha 1 chain	Secreted	0.006
FBN1	ENSG00000166147	fibrillin 1 [Source:HGNC Symbol;Acc:HGNC:3603]	Secreted	520
GPC1	ENSG00000063660	glypican 1 [Source:HGNC Symbol;Acc:HGNC:4449]	Secreted	9.5
ITGB1	ENSG00000150093	integrin subunit beta 1 [Source:HGNC Symbol;Acc:HGNC:6153]	Secreted	270
THBS1	ENSG00000137801	thrombospondin 1 [Source:HGNC Symbol;Acc:HGNC:11785]	Secreted	16000
HSPG2	ENSG00000142798	Heparan sulfate proteoglycan 2 [Source:HGNC Symbol;Acc:HGNC:3339]	Secreted	1.4
ACTG1	ENSG00000184009	Actin gamma 1 [Source:HGNC Symbol;Acc:HGNC:63261]	Secreted	4.9
LAMA5	ENSG00000130702	Laminin subunit alpha 5 [Source:HGNC Symbol;Acc:HGNC:3911]	Secreted	1.8

Table S6. Abbreviation list

Abbreviation	
BC	Breast Cancer
IDC	Infiltrating Ductal Carcinoma
ILC	Infiltrating Lobular Carcinoma
MDLC	Mixed Ductal/Lobular Carcinoma
DCIS	Ductal Carcinoma In Situ
ER	Estrogen Receptor
PR	Progesterone Receptor
HER2	Human Epidermal Growth Factor Receptor 2
TNBC	Triple negative Breast cancer
Ki-67	Marker Of Proliferation Ki-67
PIK3CA	Phosphatidylinositol 4,5-Bisphosphate 3-Kinase
TP53	Tumour Protein P53
ECM	Extracellular matrix
LGALS3BP	Galectin 3 Binding Protein
LAMB2	Laminin Subunit Beta 2
BMP1	Bone Morphogenetic Protein 1
LAMB1	Laminin Subunit Beta 1
AGRN	Agrin
LAMC2	Laminin Subunit Gamma 2
LAMB3	Laminin Subunit Beta 3
EGFR	Epidermal Growth Factor Receptor
LAMC1	Laminin Subunit Gamma 1
FBN1	Fibrillin 1
COL7A1	Collagen Type VII Alpha 1 Chain
GPC1	Glypican 1

TGFBI	Transforming Growth Factor Beta 1
CTSV	Cathepsin V
HSPG2	Heparan Sulfate Proteoglycan 2
THBS1	Thrombospondin 1
LDLR	Low Density Lipoprotein Receptor
ACTG1	Actin Gamma 1
VEGFA	Vascular Endothelial Growth Factor A
LTBP1	Latent Transforming Growth Factor Beta Binding Protein 1
FN1	Fibronectin 1
DAG1	Dystroglycan 1
ITGB1	Integrin Subunit Beta 1
MET	MET Proto-Oncogene, Receptor Tyrosine Kinase
CTSL	Cathepsin L
LAMA5	Laminin Subunit Alpha 5
SDC4	Syndecan 4
PLAU or uPA	Plasminogen Activator, Urokinase
LOXL2	Lysyl Oxidase Like 2
PI3K-Akt	Phosphatidylinositol-4,5-Bisphosphate 3-Kinase /AKT Serine/Threonine Kinase 1 pathway
NSCLC	Non-small cell lung cancer
CRC	Colorectal cancer
P	P value
HR	Hazard Ratio
CI	Confidence Interval

KEGG	Kyoto Encyclopaedia of Genes and Genomes
ELK1	ETS Transcription Factor ELK1
MYC	MYC Proto-Oncogene, BHLH Transcription Factor
MAX	MYC Associated Factor X
mTOR	Mechanistic Target Of Rapamycin Kinase
SRBP1	Insig-Srebp-Scap
PCSK9	Proprotein Convertase Subtilisin/Kexin Type 9
hAG	Anterior Gradient, Protein Disulphide Isomerase Family Member
FPKM	Fragments per kilobase of exon per million reads mapped
